# The trail making test as a screening instrument for driving performance in older drivers; a translational research

**DOI:** 10.1186/1471-2318-14-123

**Published:** 2014-11-24

**Authors:** Paul Vaucher, Daniela Herzig, Isabel Cardoso, Michael H Herzog, Patrice Mangin, Bernard Favrat

**Affiliations:** Traffic Medicine and Psychology Unit, University Center of Legal Medicine, Lausanne–Geneva, University Hospital of Lausanne, Rue Saint-Martin 26, 1005 Lausanne, Switzerland; Psychophysics Unit, The Brain–Mind Institute, EPFL, Building AAB, 1015 Lausanne, Switzerland; Traffic Medicine and Psychology Unit, University Center of Legal Medicine, Lausanne–Geneva, University of Geneva, Michel-Servet 1, 1211 Geneva, Switzerland; Department of Ambulatory Care and Community Medicine, University Hospital of Lausanne, Rue du Bugnon 44, 1011 Lausanne, Switzerland

**Keywords:** Aging, Trail making test, Fitness to drive, On-road evaluation, Psychophysics

## Abstract

**Background:**

In many countries, primary care physicians determine whether or not older drivers are fit to drive. Little, however, is known regarding the effects of cognitive decline on driving performance and the means to detect it. This study explores to what extent the trail making test (TMT) can provide indications to clinicians about their older patients’ on-road driving performance in the context of cognitive decline.

**Methods:**

This translational study was nested within a cohort study and an exploratory psychophysics study. The target population of interest was constituted of older drivers in the absence of important cognitive or physical disorders. We therefore recruited and tested 404 home-dwelling drivers, aged 70 years or more and in possession of valid drivers’ licenses, who volunteered to participate in a driving refresher course. Forty-five drivers also agreed to undergo further testing at our lab. On-road driving performance was evaluated by instructors during a 45 minute validated open-road circuit. Drivers were classified as either being excellent, good, moderate, or poor depending on their score on a standardized evaluation of on-road driving performance.

**Results:**

The area under the receiver operator curve for detecting poorly performing drivers was 0.668 (CI95% 0.558 to 0.778) for the TMT-A, and 0.662 (CI95% 0.542 to 0.783) for the TMT-B. TMT was related to contrast sensitivity, motion direction, orientation discrimination, working memory, verbal fluency, and literacy. Older patients with a TMT-A ≥ 54 seconds or a TMT-B ≥ 150 seconds have a threefold (CI95% 1.3 to 7.0) increased risk of performing poorly during the on-road evaluation. TMT had a sensitivity of 63.6%, a specificity of 64.9%, a positive predictive value of 9.5%, and a negative predictive value of 96.9%.

**Conclusion:**

In screening settings, the TMT would have clinicians uselessly consider driving cessation in nine drivers out of ten. Given the important negative impact this could have on older drivers, this study confirms the TMT not to be specific enough for clinicians to justify driving cessation without complementary investigations on driving behaviors.

**Electronic supplementary material:**

The online version of this article (doi:10.1186/1471-2318-14-123) contains supplementary material, which is available to authorized users.

## Background

The trail making test (TMT) is a neuropsychological paper-form test that was initially developed by the US army during the second world war to evaluate overall performance in new recruits[1]. During the late’40s and early’50s, two of its creators, Armitage[2] and Reitan[3], then transposed its application to assess brain injury in patients following stroke. Its ability to assess fitness to drive was first tested in 1992 for patients with closed brain injury[4] and for older drivers the following year[5]. Since then, studies have shown the TMT to be one of the best performing paper-and-pencil–based neuropsychological tests in predicting driving difficulties[6–8].

Like most neuropsychological tests, there is only a weak association between on-road evaluations and TMT performance values[7, 9]. For example, a recent study showed limitations of the TMT in correctly identifying patients deemed unfit to drive[10]. In addition, studies have so far failed to define appropriate cut-off values for the TMT-B to detect unfitness to drive[11]. These issues are crucial for many of the guidelines[12–14], including those of the American Medical Association and the Canadian Medical Association, that recommend the TMT to assess fitness to drive. The TMT is nevertheless now being used by primary care physicians who, in many countries, have assumed the responsibility of detecting unfit older drivers with some relative success[15]. Indeed, current guidelines and use of cut-off points for the TMT could lead many primary care practitioners and geriatricians to wrongly consider enforcing driving cessation when assessing fitness to drive. Given the negative consequences for home-dwelling older patients, for whom losing their driver’s license often entails important changes with negative consequences for their health[15, 16], this debate needs to be addressed more specifically. This study investigated to what extent primary care physicians and geriatricians can transpose screening results using the TMT to their patients’ hypothetical performance in an on-road evaluation.

## Methods

### Objectives

Our primary objective was to study the strength of the association of TMT with on-road performance and provide clinicians with predictive values of driving performance when screening older people in apparent healthy cognitive states. Our secondary objectives were to provide TMT-normative data for healthy older drivers, verify whether level of education is an appropriate indicator of the literacy required to perform the TMT-B, and break TMT-B down to psychophysics components known to alter with aging and cognitive decline.

### Design

This translational research was nested in two separate studies. The first was a cohort study exploring cognitive decline, driving performance, and driving cessation. The second was an explorative study in psychophysics investigating the links between cognitive decline, metabolism, and genetic factors.

### Settings

Our aim was to study a representative sample of older drivers independently of their health status. We therefore chose to test fitness to drive and on-road driving performance of older participants in a driving refresher course provided by the Swiss Automobile Club.

### Participants

In collaboration with the State Driver and Vehicle Licensing Agency and the Swiss Automobile Club, we wrote to all drivers who had reached their 70^th^ year and were residents of eastern Lausanne (fall 2011), northern Vaud and Valais (spring 2012), western Lausanne (fall 2012), and Vevey, Montreux, Aigle, and Entremont (spring 2013), inviting them to participate in a refresher course on driving competencies. In this refresher course, all participants were then offered the opportunity to participate in this study. During the spring 2013 session, older drivers were also invited to join the second part of this study investigating the psychophysics components of the TMT. To be included, participants had to hold a valid Swiss driver’s license, be aged 70 years or over, and not be institutionalized.

### TMT

The first part of the TMT measures the time participants need to connect 25 numbered circles in an ascending order (part A). In the second part (B), 13 numbers and 12 letters have to be alternately connected in their numerical and alphabetical order. Participants were notified of errors immediately and required to correct them without assistance with the clock running.

### Medical status and driving history

Older drivers were invited to volunteer for a two-hour interview to collect information on their driving history and their medical status. Visual acuity, visual field, contrast sensitivity, medication, functional mobility using the Timed Up-and-Go test (TUG)[17], the MoCA[18], average weekly distance driven, and history of accidents was some of the information we collected and then used for this study.

### Defining the healthy population for normative values

The healthy population was defined as drivers with normal optical vision (acuity ≥0.6 decimals, binocular visual field ≥140°), normal cognitive functions as per the Montreal Cognitive Assessment (MoCA ≥ 26), normal functional mobility (TUG < 13.5 sec), and no known risk of sudden blackout (history of sudden blackout, epilepsy, arrhythmia, uncontrolled diabetes, or sleep apnea), and who were not regularly or occasionally under the influence of class III medication[19].

### On-road driving evaluation

Routes were standardized for participants from the same region. They were sufficiently difficult for lapses to occur, and long enough (≈45 minutes) to assess the effects of sustained attention. Routes included urban and rural sections, secondary and principle roads and highways, simple and complex intersections, “roundabouts” (circular intersections with changing on-road priorities), traffic signals, and complex lane selections. The Swiss National Council for Road Security validated the routes. Twelve driving instructors participated in the study. They were either self-employed or were employees of the Swiss Automobile Club. They were all certified by the Swiss National Council for Road Security with a specific diploma for managing older-driver instruction. Driving instructors were blinded to the results from the psycho-medical evaluation and reported their “gestalt” evaluation of driving performance as “good” or “sufficient” for the following criteria: respecting road regulations, handling vehicle, speed adaptation, correct position on the road, comfort, behavior toward other road users, observation, and anticipation. Driving competencies were summarized as excellent (no lapse), good (lapses reported for one or two items), moderate (lapses reported for three to five items), or poor (lapses reported for six to eight items). This scoring method was verified using principle component analysis and Rash analysis thereby confirming its unique dimension (Eigenvalue = 5.1) and good fit to an overall trait (R1c = 12.2, d.f. = 14, p = 0.565).

### Literacy

To evaluate the influence of literacy on the TMT-B, an additional task was, at a later stage, developed specifically for this study: the KHE task. Participants were asked to specify which letter would come after each of three specific letters of the alphabet. As soon as a participant gave a correct answer, the next letter was provided to them. Participants were told they were to answer correctly as fast as they could. The task was timed from the moment the first reference letter was announced to the moment the third answer was provided by the participant. The duration and number of errors were then recorded. The letters used were K, H, and E, and the expected answers were “L”, “I”, and “F”.

### Psychophysical components

Over two additional two-and-a-half-hour sessions, participants in the spring 2013 session underwent a series of additional tests in our lab. A researcher, blind to the results from the TMT and the on-road evaluation, tested visual acuity (Landolt C, FrACT version 3.7 l)[20], contrast sensitivity (Gabor patch), visual backward masking (Vernier task)[21, 22], motion direction sensitivity, orientation discrimination sensitivity, biological motion, visual search (16 objects), the Simon effect, simple response time, executive functions (Wisconsin Card Sorting Test), verbal fluency, and working memory (digital forward and backward task). For further details on these tests see Additional file1.

### Statistical methods

Sample size was calculated to detect a two-fold increase in the risk of performing poorly on the driving test assuming one patient out of five would be positive to the TMT and that 20% of the participants would exhibit poor driving performance. With a significance level set at 0.05 and a power of 0.9, this required recruiting 408 participants.

We excluded patients for whom data on driving performance or TMT were unavailable. TMT was log transformed. Association to driving performance was then tested using linear regression. Driving performance was dichotomized to distinguish poorly performing drivers from all other drivers, and drivers who performed well from all others. We then defined two different cut-off values: the first to identify poor drivers with a specificity of 75%, and the second to identify good drivers with a specificity of 75%. TMT-A and TMT-B results were then combined (A and B negative to rule out, A or B positive to rule in), and predictive values measured. We then verified if this association was influenced by age, gender, education, or driving experience by the use of logistic regression. All continuous variables entered in the model were transformed to be normally distributed and range from zero to one for the fifth and ninety-fifth percentiles of the healthy population. For this analysis, missing data was completed using sequential regression multiple imputations by chained equations (50 times).

To model components of the TMT-B using psychophysics measures, we used Poisson regression with robust estimator of variance. All continuous variables were transformed to range from 0 to 1 for the twenty-fifth and seventy-fifth percentiles of the studied population. Statistical methods were defined prior to analysis and run using STATA 12, except for neural network analysis for which we used the Neural Network Toolbox 8.1 in MATLAB R2013b.

### Ethical standards

Both studies from which data was drawn were approved by the official state ethics committee for the Canton of Vaud (http://www.cer-vd.ch) under the references CE 157/2011 and CE 384/2011. An amendment was accepted in May 2013 to obtain participants’ consent to share data between the studies. All participants gave their informed consent prior to their inclusion. Both studies were performed in accordance with the ethical standards of the 2008 amended Declaration of Helsinki (Seoul).

## Results

### Population description

**Figure 1:**
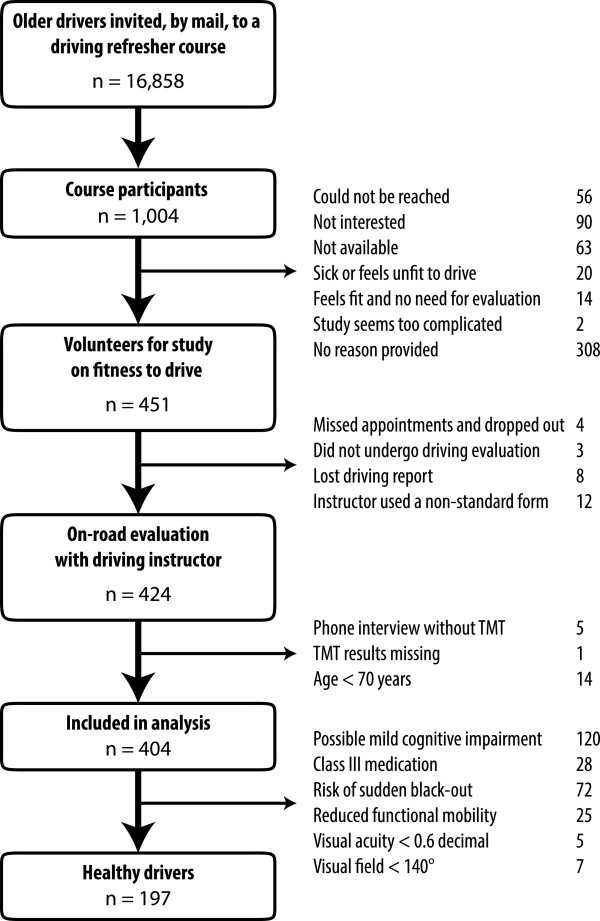
**Flow chart for the selection of older drivers.** TMT = Trail Making Test, n = sample size.

Between May 2011 and September 2013, 40.2% (404) of participants of a driving refresher course for the elderly participated in this study. Reasons for not participating are provided in Figure 1. Participants’ characteristics are described in the left column of Table 1. Forty-one of these drivers also volunteered to undergo a series of psychophysical tests at our lab.

**Table 1:** **Characteristics of volunteer, older, home-dwelling drivers and of those considered as healthy drivers**

Determinants	All study participants (n = 404)	Healthy* (n = 197)	“Unhealthy”* (n = 207)	P-value^‡^
	Median (p5–p95) / % (n)	Median (p5–p95) / % (n)	Median (p5–p95) / % (n)	(Chi^2^or ***T***-test)
Age (years)	75.4 years (70-84)	74.8 (70–83)	75.8 (71–85)	P = 0.025
Gender (% male)	62.6% (n = 253)	52.8% (n = 104)	72.0% (n = 149)	P < 0.001
Education (% <12 yrs)	26.2% (n = 106)	23.3% (n = 46)	29.0% (n = 60)	P = 0.198
**Vision**				
Visual acuity (decimal)	1.0 dec (0.6–1.2)	1.0 (0.8–1.2)	1.0 (0.6–1.2)	P = 0.003
Visual field (°)	180° (150–200)	180 (160–200)	180 (150–200)	P = 0.106
Contrast sensitivity (log[CS])†	1.72 log(CS) (1.6–1.76)	1.72 (1.6–1.76)	1.72 (1.6–1.76)	P = 0.429
**Functional mobility**				
Timed up-and-go test – TUG (sec)	8.0 sec (6.0–13.0)	8.0 (6.0–12.0)	8.4 (6.0–14.8)	P < 0.001
**Cognitive state**				
MoCA (points [0-30])	27 points (21–30)	28 (26–30)	25 (20–29)	P < 0.001
MoCA_mod_ (points [0-29])	26 points (20–29)	27 (24–29)	24 (19–28)	P < 0.001
**Driving**				
Distance driven per week (km)	200 km (50–500)	200 (40–500)	200 (50–500)	P = 0.832
History of accidents during the previous 2 years				
*All types with material damage*	26.0% (n = 105)	25.4% (n = 50)	26.6% (n = 55)	P = 0.785
*Responsible for damage to others*	7.4% (n = 30)	6.6% (n = 13)	8.2% (n = 17)	P = 0.536
*Injured*	1.2% (n = 5)	0% (n = 0)	2.4% (n = 5)	P = 0.028
On-road driving performance				P = 0.605
*Excellent*	47.0% (n = 190)	46.2% (n = 91)	47.8% (n = 99)	
*Good*	27.0% (n = 109)	27.9% (n = 55)	26.1% (n = 54)	
*Moderate*	20.5% (n = 83)	21.8% (n = 43)	19.3% (n = 40)	
*Poor*	5.4% (n = 22)	4.1% (n = 8)	6.8% (n = 14)	

*The healthy population was defined as drivers with normal optical vision, no cognitive impairment (MoCA ≥ 26), normal functional mobility (TUG < 13.5 sec), no known risk of sudden blackout, and without class III medication affecting driving performance. † MARS contrast sensitivity was not collected from the start of the study and was therefore available for only 158 participants, of whom 76 were healthy. ‡ P-Values are for comparing healthy participants to “unhealthy participants”. CS = contrast sensitivity, MoCA = Montreal cognitive assessment, MoCA_mod_ = Modified MoCA (without TMT or education level).

### Reference values for the healthy population

One hundred and ninety-seven participants (48.8%) were considered to be healthy. Reasons for excluding the remaining 207 are provided in Figure 1. Compared to other drivers, healthy drivers were younger, were more likely to be female, and were less likely to have been involved in an accident involving injury during the previous two years (Table 1). Half of the healthy drivers took less than 42 seconds to perform the TMT-A, and less than 94 seconds to perform the TMT-B (Table 2). Our observations reveal that independently of age or education TMT-A and TMT-B durations show very important variations in healthy older drivers; normal values ranged from simple to triple. We observed a slight increase in the duration of the TMT for drivers aged 80 and upward compared to other older drivers. The mean difference was of 8.4 seconds for the TMT-A (R^2^ = 0.038, p = 0.006) and 31.7 seconds for the TMT-B (R^2^ = 0.061, p < 0.001). Lower education level and gender were associated to TMT-B but not TMT-A. Difference related to gender seemed to arise from a minority of male participants with very slow performances. As for age and education, when observing the probability distribution of TMT-B values, we noticed an overall shift of values toward slower execution times. This supports the hypothesis that cognitive decline affects performance for all drivers even in the absence of motor- or cognitive disorders and that difficulties with the alphabet might need to be accounted for.

**Table 2:** **Normative data for healthy, home-dwelling, older drivers (n = 197)**

	TMT-A (seconds)			TMT-B (seconds)		
	Mean	SD	Median [p5-p95]	Mean	SD	Median [p5-p95]
**Gender**						
Female (n = 93)	43.8	14.6	40 [26-68]	94.5	31.2	91 [54-157]
Male (n = 104)	45.6	17.9	40 [27-80]	117.6	59.0	98 [56-204]
**Age**						
<75 years (n = 101)	43.2	14.6	40 [26-67]	103.3	47.8	91 [53-187]
75 to 79 years (n = 61)	43.3	14.9	40 [24-72]	97.6	34.2	90 [58-172]
≥ 80 years (n = 35)	51.7	22.0	44 [29-113]	132.8	65.3	129 [66-214]
**Education level**						
> 12 years of schooling (n = 151)	44.7	16.4	40 [26-72]	101.6	45.8	90 [54-182]
≤ 12 years of schooling (n = 46)	44.8	16.9	42 [28-76]	123.8	56.3	102 [70-214]
**Entire healthy population**	44.8	16.5	41 [26-74]	106.7	49.2	93 [54-187]

For this analysis designed to provide normative values for healthy older adults, we excluded measures from patients with health conditions that might have affected their performance. Therefore, normative data is provided for older drivers with normal optical vision, no cognitive impairment (MoCA ≥ 26), normal functional mobility (TUG < 13.5 sec), no known risk of sudden blackout, and without class III medication affecting driving performance. TMT = trail making task, SD = standard deviation.

### Literacy versus years of education

Seventy consecutive participants completed the KHE task. The main result was the time needed to provide correct responses and this ranged from 2.8 seconds to 25 seconds with a median at 6.8 seconds. At least one error was made by 21.7% of participants. On average, making an error increased the duration of the task by 5.6 seconds (CI95% 2.8 to 8.5, p < 0.001). KHE task durations of 12 seconds or more were considered as positive (n = 9). KHE performed better in predicting the number of seconds required to complete the TMT-B than did level of education (R^2^ = 0.023 vs. R^2^ = 0.006, likelihood ratio test p < 0.001). From our regression analysis, to adjust for difficulties with the alphabet the overall TMT-B values should be reduced by 25% for those with a positive KHE task (≥12 seconds).

### Psychophysical properties of the TMT-B

A subset of 45 drivers underwent a series of psychophysical tests (Table 3). TMT-B performance was significantly correlated to contrast sensitivity (IRR = 1.12, R^2^ = 0.075, p = 0.019), motion direction (IRR = 1.17, R^2^ = 0.125, p = 0.001), orientation discrimination sensitivity (IRR = 1.17, R^2^ = 0.091, p < 0.001), working memory (IRR = 1.33, R^2^ = 0.109, p = 0.003), verbal fluency (IRR = 1.18, R^2^ = 0.074, p = 0.027), and literacy (IRR = 1.23, R^2^ = 0.146, p = 0.001). Interestingly, TMT-B performance was not associated to visual acuity (IRR = 1.03, R^2^ = 0.004, p = 0.528) or to mental flexibility as measured by the Wisconsin Card Sorting Test (IRR = 1.11, R^2^ = 0.035, p = 0.168).

**Table 3:** **Modeling the psychophysical components of the TMT-B (n = 45)**

	TMT-B	
	IRR^†^[CI95%]	R^2^
**Socio-demographic**		
Age (years)	1.05 [0.91 to 1.20]	0.006
Gender (male)	1.14 [0.89 to 1.45]	0.016
Education (years of schooling)	0.91 [0.79 to 1.04]	0.150
Literacy (KHE [sec])	1.23 [1.09 to 1.39]*	0.146
**Optical tests**		
Visual acuity (FrACT arc minutes)	0.97 [0.88 to 1.06]	0.004
Contrast sensitivity (75% threshold Gabor patch)	1.12 [1.02 to 1.22]*	0.075
**Neuropsychological tasks**		
Visio-spatial search (TMT-A [sec])	1.25 [1.10 to 1.41]*	0.157
Mental flexibility (Perseverative errors WCST [%])	1.11 [0.96 to 1.29]	0.035
Working memory (Digit backwards [n°])	0.75 [0.62 to 0.91]*	0.109
Verbal fluency ([animals + fruit–veg.] /2 [n°/min])	0.85 [0.73 to 0.98]*	0.074
Functional mobility (timed up-and-go test - TUG [sec])	1.11 [0.99 to 1.11]	0.047
**Computed tasks**		
Visual processing (Vernier’s Task)		
*Vernier offset (ms)*	1.12 [0.93 to 1.34]	0.023
*Vernier duration (ms)*	1.12 [0.90 to 1.40]	0.015
*Masking effect without shine through SOA 5 (ms)*	1.11 [0.97 to 1.27]	0.035
Simple response time (ms)	1.11 [0.93 to 1.32]	0.029
Visual search (RT [ms])	1.12 [0.96 to 1.32]	0.040
Motion direction sensitivity (% dots)	1.17 [1.06 to 1.29]*	0.125
Orientation discrimination sensitivity	1.17 [1.09 to 1.25]*	0.091
Biological motion	0.95 [0.80 to 1.13]	0.005
Simon’s effect (Δt [ms])	1.03 [0.86 to 1.25]	0.002

*Significant at p < 0.05. † Incidence risk ratio was measured using Poisson regression. Continuous values were transformed to be normally distributed and have a range of 0 to 1 from the twenty-fifth to the seventy-fifth percentile of the population. FrACT = Freiburg Visual Acuity and Contrast Test, IRR = Incidence-rate ratio, R^2^ = Coefficient of determination, SOA = Stimulus-onset-asynchrony.

### On-road evaluation

**Figure 2:**
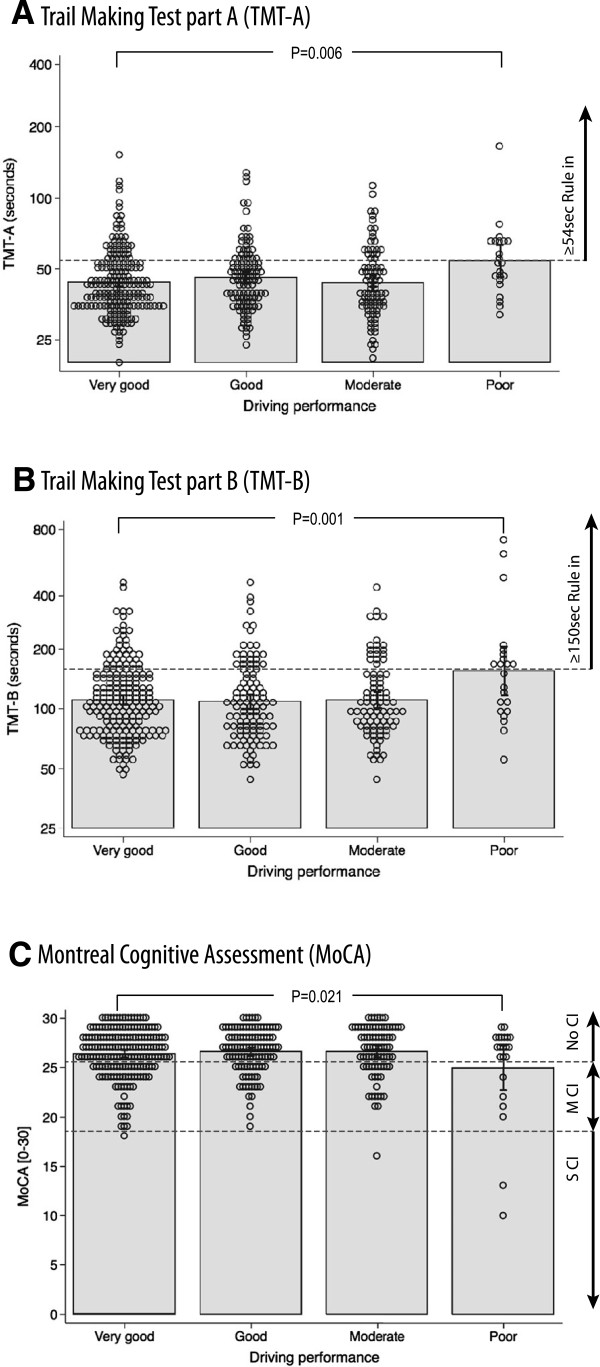
**Driving performance and results from tests including TMT.** Open dots correspond to each individual observation, grey blocks’ upper borders correspond to mean observed TMT duration on a log scale. Interval bars represent 95% CI. P-values correspond to F statistics comparing poor drivers to excellent drivers. For figures **A** &**B**, doted lines represent the threshold (TMT-A ≥54 sec; TMT-B ≥150 sec) from which TMT duration can evoke unfitness to drive. For figure **C**, doted lines represent threshold of the MoCA to differentiate drivers without cognitive impairment (NoCI), those with mild cognitive impairment (MCI), and those with severe cognitive impairment (SCI).

Of the 404 older drivers, 190 (47.0%) were considered to be excellent drivers, 109 (27.0%) good drivers, 83 (20.5%) moderate drivers, and only 22 (5.4%) poor drivers. TMT results show that many excellent drivers have poor results on this test (Figure 2). Nevertheless, independently of age, gender, and education level, compared to other drivers those that performed poorly on the on-road evaluation took 22.2% (CI95% 0.4 to 48.7, p = 0.045) more time to perform the TMT-A and 63.9% (CI95% 14.8 to 134.2, p = 0.007) more time for the TMT-B. The TMT’s ability to correctly classify those with poor driving performance was above chance for both TMT-A (AUC = 0.668, CI95% 0.558 to 0.778) and TMT-B (AUC = 0.662, CI95% 0.542 to 0.783). Older drivers were then categorized into three groups. Those who had a TMT-A < 35 sec and a TMT-B <80 sec were ruled out as being unfit to drive (13.1% of drivers), those who had a TMT-A ≥54 sec or a TMT-B ≥150 sec were ruled in as been potentially unfit to drive (35.1%), and the remaining drivers (51.8%) remained in a grey zone. Not a single driver from the fit group was evaluated as a poor driver whereas fourteen of the 148 “unfit” drivers (9.5%) were. We observed a threefold increase in the risk of been a poor driver if TMT-A ≥54 sec orTMT-B ≥150 (CI95% 1.3 to 7.0; p = 0.007). Relying on the TMT alone, we would nevertheless need to send approximately one participant out of three for an on-road evaluation. Of each ten patients who would then undergo the on-road evaluation, only one would be considered to be a poor driver (sensitivity = 63.6%, specificity = 64.9%, PPV = 9.5%, NPV = 96.9%, PLR = 1.81, NLR = 0.56). Other than the TMT, cognitive impairment, as measured by the modified MoCA, and driving experience were also associated to on-road driving performance (Table 4). However, the MoCA, which includes a modified TMT, was only associated to poor driving performance for those with severe cognitive impairment but not for those with mild cognitive impairment (Figure 2C and Table 4). The model for driving performance including TMT-A, age, timed up-and-go test, and distance driven per week (Table 4; Model A) showed only TMT-A and distance driven to be related to driving performance. The same was observed when modeling TMT-B including education level (Table 4: Model B). Furthermore, including these factors in neural network modeling did not perform any better than using the TMT alone in identifying drivers with poor driving performance (sensitivity = 52.8%, specificity = 43.3%, PPV = 53.0%, NPV = 97.5%).

**Table 4:** **Crude and adjusted odds of having poor driving competencies (N = 404)**

Determinants	Crude		Model 1-TMT-A		Model 2-TMT-B	
			R^2^ = 0.081		R^2^ = 0.107	
	OR [CI95%, p-value]	R^2^	OR_adj_	P-value	OR_adj_	P-value
**Neuropsychological tasks**						
TMT-A*	5.1 [1.5 to 17.4, p = 0.010]	0.036	3.8	0.041	-	-
TMT-B*	7.6 [2.2 to 25.8, p = 0.001]	0.058	-	-	6.3	0.005
MoCA		0.043				
NoCI (MoCA ≥ 26)	Reference					
MCI (MoCA < 26 & ≥19)	0.60 [0.20 to 1.8, p = 0.368]					
SCI (MoCA < 19)	16.7 [2.2 to 127, p = 0.006]					
MoCAmod*†	4.4 [1.4 to 13.7, p = 0.010]	0.034	-	-		
**Socio-demographic**						
Age*	1.6 [0.43 to 6.0, p = 0.468]	0.003	0.85	0.821	0.83	0.797
Gender (female)	2.1 [0.88 to 5.0 p = 0.093]	0.016	-	-	-	-
Education (<12 yrs)	0.81 [0.3 to 2.3, p = 0.701]	<0.001	-	-	0.59	0.349
**Functional mobility**						
Timed up-and-go test (TUG)	3.2 [0.9 to 11.1, p = 0.066]	0.020	2.4	0.178	2.2	0.210
**Driving experience**						
Distance driven per week*†	5.4 [1.6 to 17.4, p = 0.004]	0.043	4.1	0.022	3.9	0.031
**Vision**						
Visual acuity*†	1.5 [0.44 to 5.3, p = 0.514]	0.002	-	-		

*Odds ratio was measured using logistic regression. Continuous values were transformed to be normally distributed and have a range of 0 to 1 from the fifth to the ninety-fifth percentile of the population. † Scale was inverted for high scores to represent worsening conditions. We used multiple imputations (50) to replace missing data (TUG, n = 10; distance per week, n = 2; visual acuity, n = 3). MCI = mild cognitive impairment; MoCA = Montreal Cognitive Assessment, MoCAmod = Modified Montreal Cognitive Assessment (First subtask similar to TMT-B omitted), NoCI = No cognitive impairement; R^2^ = McFadden's pseudo R-squared coefficient of determination; SCI = severe cognitive impairment.

### History of motor vehicle collisions

One hundred and sixty-seven drivers reported having had a motor vehicle collision (MVC) during the past two years (41.3%). Those who either had a TMT-A ≥54 seconds or a TMT-B ≥150 seconds were more likely to have had a shorter period without MVC than other drivers (HR = 1.48, CI95% 1.06 to 2.06, p = 0.022).

## Discussion

### Overview of results and clinical applications

This study shows that cognitive decline in the absence of disease affects the TMT and driving performance. Decline mainly concerns drivers aged 80 years or more. Using the TMT for screening purposes below that age seems unjustified unless there is an underlying known cause of cognitive decline. We also advise not to rely on an age-specific percentile to define cut-off points of abnormality given that this can lead to natural cognitive decline not being accounted for. The same applies for education level as it neglects underlying cognitive deficits that would have also affected scholarship. Instead we suggest verifying patients do not have difficulties with the alphabet. We suggest TMT-B results to be invalid for those who require 12 seconds or more to perform the KHE test. Under these conditions, our study provides clinicians with a simple rule in interpreting TMT results when screening for unfitness to drive. Effects of cognitive decline on driving can be ruled out for those who can perform the TMT-A in less than 35 seconds, and the TMT-B in less than 80 seconds. On the other hand, negative consequences of cortical dysfunction for driving performance can be suspected for those with a TMT-A ≥54 seconds or a TMT-B ≥150 seconds. These drivers are three times more at risk of being poor drivers. However, if we were to have all these people cease driving, we would uselessly reduce the mobility of nine out of ten positive patients. This is absolutely to be avoided, as reducing mobility is known to affect patients and have important negative consequences on their health[15, 23, 24].

Our results show that the psychophysical functions evaluated by the TMT are those that are indeed most useful for driving. In other words, the TMT is affected by reduced performance on basic visual tasks that are deemed essential for driving. Why, then, is TMT performance only weakly correlated with on-road performance? The similarly bad performance of the MoCA suggests this is not due to the lack of sensitivity of the TMT. Our results even suggest that the TMT does better than the MoCA in classifying poor performing drivers from other drivers when screening older drivers. The TMT is known to perform better in detecting poor performing drivers compared to the mini mental state exam (MMSE). The important load of memory tests within these batteries of tests might affect their validity in predicting on-road events. Memory has indeed been shown to have little to do with driving performance[7]. On the other hand, the TMT is a more precise indicator of reduced visual processing speed[25, 26]. We suggest that older drivers may be well aware of their visual limitations related to cognitive decline and have had time to adapt their behavior so that they are not perceivable during the on-road evaluation. In older drivers, tactical and strategic compensations have been shown to reduce the risks of accident[27]. The underlying mechanisms of these compensations remain unknown and the compensations are, thus, difficult to evaluate clinically. When investigating cognitive decline, we therefore encourage physicians to confirm unfitness to drive with an on-road evaluation. Occupational therapists are the best placed, in collaboration with a driving instructor, to address this problem[28].

### Comparison to previous studies

Our results are very similar to those of Classen et al.[29] who used an arbitrary cut-off point set at TMT-B > 180 sec and found an OR = 2.5 of failing an on-road test. Mazer et al.[30] used a different arbitrary cut-off point of three or more errors during the TMT-B in patients with stroke and found an OR of 6.0 to be judged as a bad driver, using a 43-item assessment form filled in by an occupational therapist. We have reasons to believe that the association of the TMT-B to road accidents could even be weaker as Ball et al.[31] found an OR of 1.21 and Marottoli et al.[32] an HR of 1.42 and Rozzini et al.[33] an OR of 2.3. All these results show that the TMT does not clearly distinguish poor drivers from others. When comparing our results to those of other tests, the TMT does just as badly in distinguishing good from poor drivers as any other test, including the UFOV[7], or combination of determinants such as the 4C[34]. It has also been shown that 40% of drivers with severe cognitive impairment are considered as competent drivers during on-road evaluations[35]. This suggests that the TMT’s lack of precision is not due to the nature of the test itself, but more to the complexity of the ways in which older drivers adapt their behavior to their condition and the fact that they can perform well even if they are affected by cognitive decline.

### Limitations

The studied population was not randomly sampled from the general population and corresponds to approximately 6% of all older drivers from four regions. Nevertheless, the prevalence of accidents involving injury was very similar to that observed in the general population[36], and the prevalence of minor cognitive impairment was not lower than that usually expected[37]. Finally, in Switzerland, from the age of 70 years onwards, drivers are requested to have a physician assess their fitness to drive every two years. We therefore believe this sample to be representative of patients without severe cognitive impairment attending their primary care physician for their compulsory evaluation of fitness to drive.

Another limitation is related to the debate over whether on-road evaluation is, or is not, the ‘gold standard’ of driving performance. In other words, is there a strong link between on-road evaluation and road accidents? Keall and Frith[36] showed that drivers of 80 years or more who fail an on-road driving test had an increased risk of 1.7 times (CI95% 1.3 to 2.2) of being involved in a crash involving injury in the following two years. This cannot be considered as a strong link but is nevertheless of the same magnitude as the increased risk observed for drivers with 0.08% blood alcohol concentrations. Conversely, this also means that in Keall and Frith’s study, 98.8% of drivers who failed the on-road test were not involved in an accident involving injury and would therefore have been unjustly prevented from driving had their licenses been withdrawn. This is nevertheless the cost that our society is ready to pay for road safety. Contrarily to those who drink and drive, older drivers do not choose to become impaired. We therefore must always keep in mind that older drivers carry the burden of this sacrifice and should be treated with the highest respect and regard for agreeing to do so.

## Conclusion

Our results do not support the use of the TMT as a single measure in deciding whether or not an older driver is unfit to drive. A discussion on the potential impact of cognitive decline on driving performance should be initiated for those with TMT-A ≥54 seconds or those with TMT-B ≥150 seconds. When driving difficulties are identified, efforts should be made to have elderly drivers themselves make the decision to give up driving. Physicians are well placed to encourage them in this process and to help finding alternative solutions to maintaining the elderly’s mobility[38].

## Electronic supplementary material

Additional file 1:**Description of psychophysical tests.** This pdf file provides details on the methods used to measure underlying psychophysical functions potentially related to the Trail Making Task. (PDF 1 MB)

## Abbreviations

FrACT: Freiburg visual acuity and contrast test
IRR: Incidence-rate ratio
MCI: Mild cognitive impairment
MoCA: Montreal cognitive assessment
R^2^: Coefficient of determination
SCI: Severe cognitive impairment
SOA: Stimulus-onset asynchrony
TMT: Trail making test
TMT-A: First part of TMT
TMT-B: Second part of TMT
TUG: Timed up-and-go test.
