# Supplementary material for: The trail making test as a screening instrument for driving performance in older drivers; a translational research
Source: BMC Geriatr. 2014 Nov 24;14:123. doi: 10.1186/1471-2318-14-123 (PMC4256796; doi:10.1186/1471-2318-14-123)
Supplement: Supplementary file 1 — Additional file 1:Description of psychophysical tests. This pdf file provides details on the methods used to measure underlying psychophysical functions potentially related to the Trail Making Task. (PDF 1 MB) [file 12877_2014_1058_MOESM1_ESM.pdf]

DESCRIPTION OF PSYCHOPHYSICAL TESTS

This file provides details on the methods used to measure underlying psychophysical functions potentially related to the Trail Making Task.

1. Visual acuity

FrACT is a computerized VA test presenting Landolt-C optotypes (**fig. 1**).

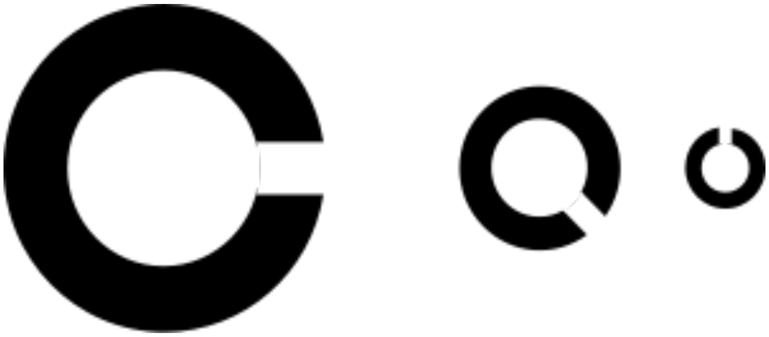

Figure 1: Landolt rings as used in the Freiburg Visual Acuity Test.

This test has been developed and described in detail by Bach.[1] It has been validated in various studies.[2,3] In our study, participants were tested at a distance of 5 m. In each trial, the size of optotype was adaptively determined following the best-PEST algorithm.[4] Consequently, step sizes are initially quite large, but become smaller when the algorithm homes in on the VA threshold. Although FrACT can optionally display the Landolt rings in eight different gap positions, we used only the four cardinal positions, because previous experience had shown this to be less confusing for elderly patients. The operation of the response box by the patients themselves often proved difficult, so the examiner operates it. During the training phase, the examiner evaluates the participant's behavior. When right–left confounds are observed, the patient is instructed to point in the perceived direction. Only one Landolt ring is displayed at a time, and the subjects' verbal response is entered into the computer by the examiner. The participants always have to report a gap direction, even if based on "best guess". This requires gentle coaxing in the training phase.

2. Contrast sensitivity

Contrast sensitivity was determined by a two-interval forced-choice procedure similar to Lahav et al.[5] We measured the Gabor contrast detection thresholds at which observers reached 75%

correct (80 trials) with an adaptive staircase method (PEST) [6] and maximum-likelihood estimation of the psychometric function parameters. The Gabor had a spatial frequency of 4 cycles per degree and appeared either in the first interval within a red ring or in the second interval within a green ring (**fig. 2**). Observers sat at 200cm distance in a dimly illuminated room. The outcome of interest is the Michaelson contrast sensitivity threshold.

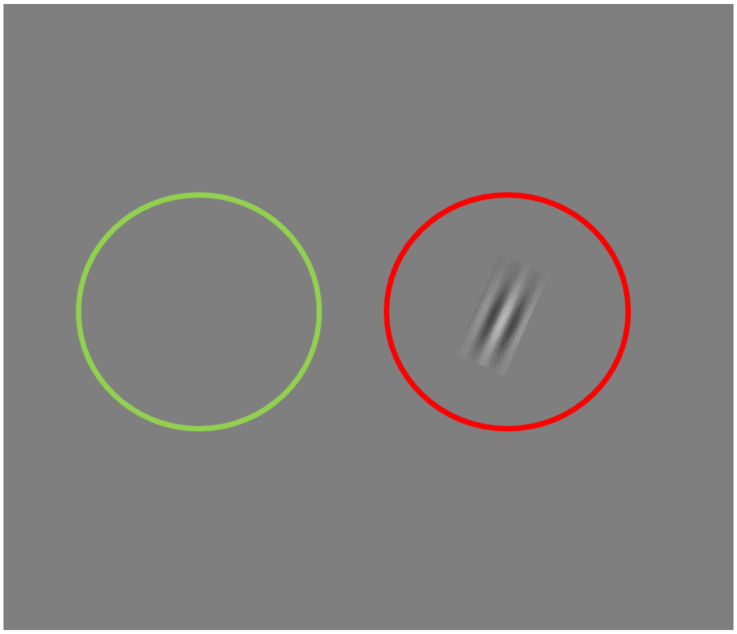

Figure 2: Gabor stimulus as used in the contrast detection paradigm.

3. Vernier acuity & visual backward masking

This task has been frequently used in our lab, and measures cortical rather than retinal processing.[7,8] Stimuli are presented from a distance of 500cm in a dimly illuminated room. The stimuli are white on a black background (**fig. 3**).

In a first step, we present vernier stimuli which consist of two vertical bars which are offset in the horizontal direction for 150 arcsecs first. In each trial, the vernier offset direction is chosen randomly. Participants indicate the offset direction of the lower bar compared to the upper bar (binary task). Errors are indicated by an auditory signal. For each observer, we determine the individual vernier duration (VD) to reach 75% correct responses using a staircase procedure (for details, see Herzog et al., 2004)[8]. In the second step, we use the individual vernier offset size as defined in the first step. The vernier is here followed by a variable inter-stimulus interval (ISI), i.e., a blank screen, and then a grating for 300 ms. The grating consists of either 5 or 25 verniers without offset of the same length as the target vernier (referred to as BM5 or BM25 respectively, BM for backward masking).

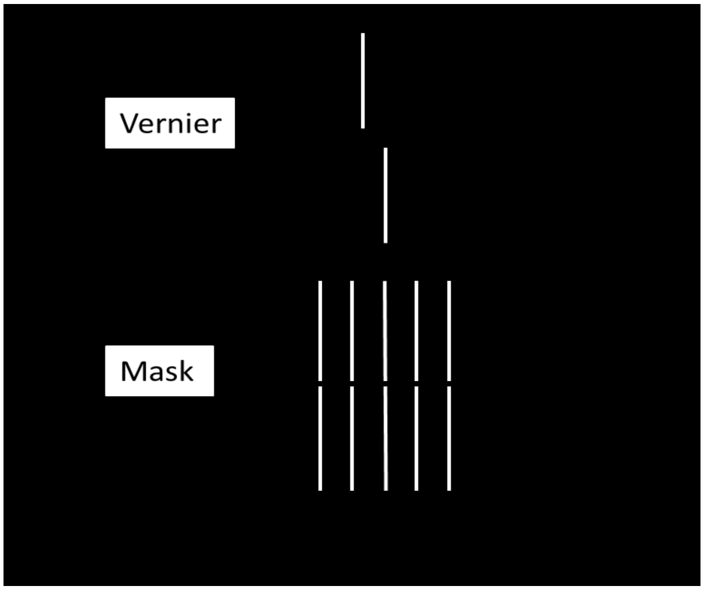

Figure 3: Vernier and backward masking task.

The five element grating leads to stronger masking than the 25 element grating even though the five element grating is contained in the 25 element grating.[9] This difference in masking strength indicates that a substantial part of the masking power is not of retinal origin because retinal processing is mainly determined by the sheer amount of light, e.g., the number of grating elements. We vary the Interstimulus Interval (ISI), i.e., the duration of a blank screen between Vernier disappearance and grating onset, for which observers reach 75% correct responses, using again the PEST staircase procedure.[6]

4. Motion direction sensitivity

This task measures global motion perception, and the stimuli and procedure are based on a paper by Roudaia et al.[10] Participants are seated 200cm in front of the screen and have to judge the general motion of dot patterns. On each trial, the pattern displays dots, which are either displaced uniformly either to the right or to the left (targets), or move independently from each other (distractors). This is illustrated in **fig. 4**. The percentage of target dots as compared to the amount of distractor dots varies randomly according to a staircase procedure (PEST).[6] The target starting value is 10%.

The experiment starts after a 30s period of adaptation, during which subjects fixate the black screen. Each trial begins with a small white fixation cross in the center of the screen. It remains on for 3 s and then disappears for 0.25 s before the appearance of the first pattern. Both patterns are presented for a duration of 100 ms separated by an ISI, during which time the entire screen is black. Subjects are instructed to fixate on the fixation point at the beginning of each trial. After the two patterns disappear, they are asked to press the green button in their right hand when they saw the dots move to the right or to press the red key in their left hand when they saw the dots

move to the left. After the subjects' key response is recorded, the fixation point reappears, indicating the start of the next trial. The outcome of interest is the measured threshold expressed as proportion of moving dots.

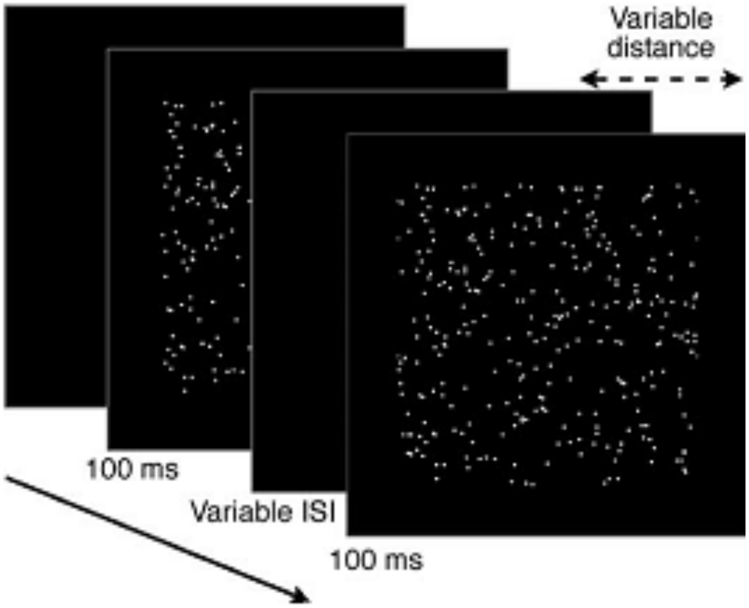

Figure 4: Global motion task sample display

5. Orientation sensitivity

This is an adapted version of the orientation discrimination task as used by Tibber et al.[11] Participants are seated 200cm from the computer screen, and required to decide whether the target stimulus is oriented clockwise (CW, right green button) or anticlockwise (ACW, red left button). The Gabor patch (Michelson contrast of 34%, average luminance of 21 cd/m2, spatial frequency of 4 cyc/deg) is illustrated in **fig. 5**. Auditory feedback is given when an incorrect response is registered. The outcome of interest is the measured threshold expressed as radial angle from the vertical line.

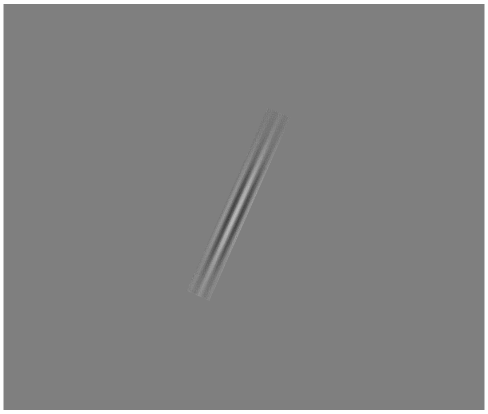

Figure 5: Orientation discrimination task stimuli (clockwise)

6. Biological motion

This task measures biological motion perception, and is based on a paper by Pilz et al.[12] Participants are seated 60 cm form the screen, and have to indicate the walking direction of a point-light walker (**fig. 6**).

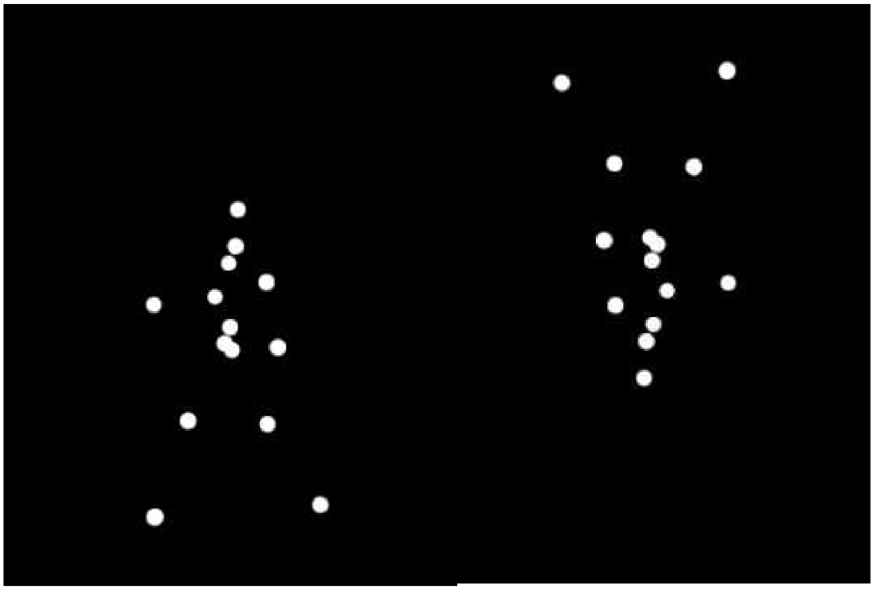

Figure 6: Biological motion task sample display

The walker does not translate across the screen, but rather appears to walk in place as if on a treadmill. The animated walker consists of 11 dots that simulated points on the head, near the shoulder, both elbows, both wrists, the hip, both knees and both ankles. Each subject was seated in a darkened room, and viewed the stimuli binocularly with a chin/forehead rest stabilizing the subject's head at a distance of 60 cm from the screen. On each experimental trial, subjects saw a side-view of a point light walker. The walker's direction of motion was either rightward or leftward, and the walker was either presented upright or inverted. Stimulus duration was either 200 or 800ms. Each subject performed 50 trials per stimulus duration (200, 800ms) or condition (upright, inverted), resulting in a total of 200 trials. All conditions (2 orientations × 2 durations) were randomly intermixed for each subject. Upright and inverted walkers are presented in separate blocks. On each trial, subjects have to decide whether the walker is walking towards their left ('S'-key) or right ('L'-key) by pressing the respective button on a standard computer keyboard. Prior to the start of the main experiment, each subject completes two blocks – one for upright point-light walkers and another for inverted – of 20 practice trials for stimuli presented. The primary output of interest is the proportion of correct answers for the upright 800 ms stimuli.

7. Visual search

The task is a modified version of Theeuwes & Kooi' visual search task.[13] Participants are tested in a dimly-lit room and seated 200 cm from the computer screen. They have to search for a green horizontal line segment within an array of distractors (**fig. 7**).

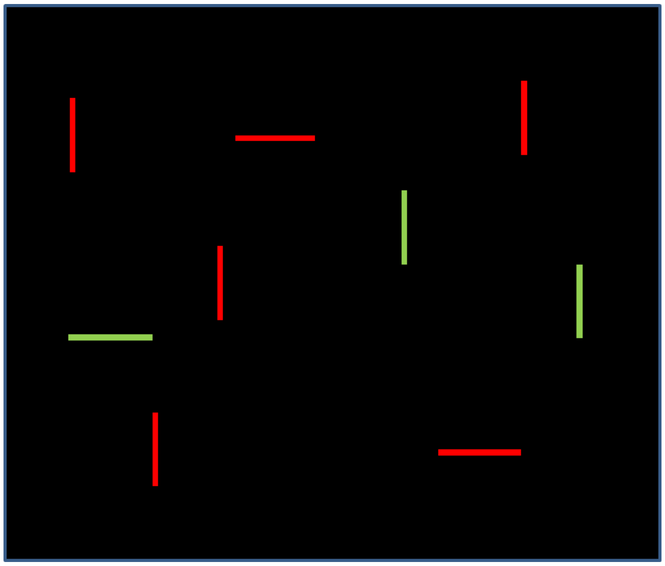

Figure 7: Visual Search task sample display (9 line segments)

The display elements are presented on a uniform black background. They consist of green or red line segments. The stimulus field consists of 4, 9 and 16 line segments with a stimulus length of 1600 arcsecs and 450 arcsecs line width. In order to detect the target, a conjunction of features (color and orientation) was required. Subjects responded 'yes' if a target was present by pressing the green button in their right hand and 'no' if it was not by pressing the red button with their left hand. On trials in which a response error is made or no response is given, the computer beeps to inform the subjects that they had committed an error. Consecutive trials are separated by a blank inter-trial interval of 1500 ms. Subjects perform a single block of 120 trials in which there are equal numbers of target present and target absent trials. Both speed and accuracy are measured. The primary outcome of interest is the average speed for correct answers for trials with 16 segments.

8. Simon effect

The Simon-task was originally developed in the auditory domain to measure impact of a task-irrelevant stimulus feature on response conflict.[14] A very good overview over the mechanisms involved in producing this effect can be found in Hommel.[15] In our study, we use a modified version of a visual Simon-task found to be sensitive to aging effects.[16] Subjects are seated 200cm observation distance to the computer screen, and are subsequently presented with arrows and

instructed to respond with the same hand as indicated by the direction of the arrow head (respond with right hand to a right pointing arrow; respond with left hand to a left pointing arrow, **fig. 8**).

There are two trial types. Congruent trials consist of the presentation of a right pointing arrow positioned at far right of the screen, or a left pointing arrow positioned at far left of the screen. Incongruent trials consisted of the presentation of a right pointing arrow positioned at far left or a left pointing arrow positioned at far right of the screen. The arrow stimuli are presented one at a time with each arrow displayed on the screen for 100 ms, then immediately followed by a blank screen for 500 ms. Subjects' responses are recorded for a maximum of 3000sec after stimulus presentation. Errors, which consist of an opposite hand response to the direction of the arrow head, are also recorded. Feedback is not provided. The principle outcome of interest is the average difference in time to response for correct answers between congruent and incongruent conditions (milliseconds).

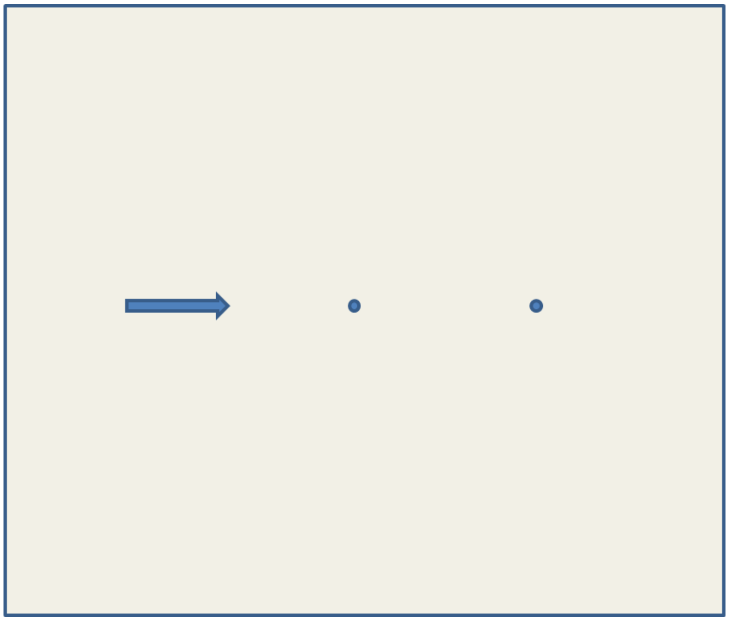

Figure 8: Visual Simon task sample display (incongruent condition)

9. Auditory volume sensitivity

During each trial, sequences of pure tones of 1kH are presented at increasing volume, ranging from -20DB to 80 DB maximum. The ISI of each trial varied randomly to prevent participants from predicting the onset of a trial. The task of the participants was to determine when they could hear the tone by pressing SPACE-BAR on the keyboard. Auditory threshold (the quietest tone a person is able to detect) is calculated as an outcome variable (dB).

10. Auditory pitch sensitivity

During each trial, two pure tones of 100 ms with 20 ms cosine rise/fall were presented in two intervals 500 ms apart. Tones were presented at 80 dB. One of the tones had a frequency of 1 kHz, whereas the other had a frequency of 1 kHz Δf (with Δf being 1, 4, 16, or 64 Hz). The reference tone of 1 kHz was presented randomly in the first or the second interval. In each trial, one of the four levels of Δf was chosen randomly. All levels of Δf were presented equally many times. The task of the participants was to determine whether the tone with the higher frequency (1 kHz +Δf) was presented in the first or the second interval. Participants responded by pressing one of two buttons. Visual feedback (the words "Correct" and "Wrong" in green and red letters, respectively) was provided after each response. The outcome of interest was the auditory pitch threshold expressed in Hz.

11. Simple response time

This task was a modified version of the classical Hick-paradigm.[17] Participants are seated at 200cm distance to the computer screen and are instructed to press a button immediately after a white square (3 arc degrees size) appears on the screen on black background (**fig. 9**).

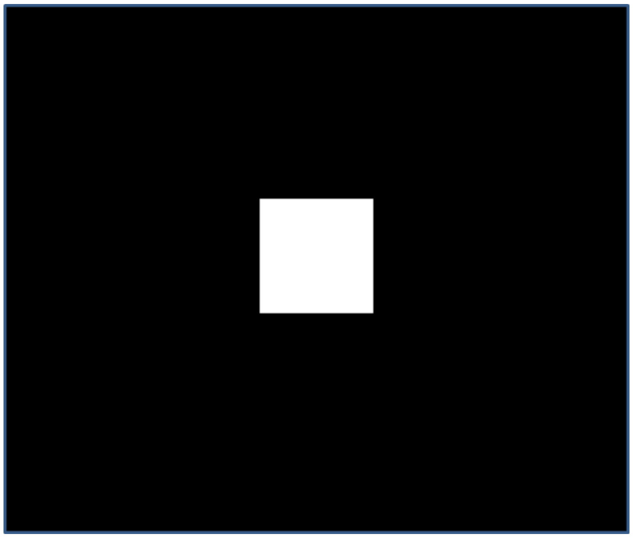

Figure 9: Simple RT task sample display

The inter-trial interval is varied randomly to prevent participants from predicting when a square would appear, but with a minimal ISI of 1500ms. RT is used as a measure of behavioral response/speed of information processing. The outcome of interest is the average response time (ms) observed over 60 trials.

12. Wisconsin Card Sorting Test

We administered a computerized version of the WCST,[18] which contains 64 cards that are displayed at the top of the computer screen four at a time (**fig. 10**). Each card contains items that differ from the other three cards on three dimensions, namely in shape (crosses, triangles, circles or stars), number (1-4 items) and colour (red, blue, yellow, green). A fifth card is then presented on the bottom of the screen, and participants need to match the fifth card according to the shape, number of items or colour by clicking on one of the four cards displayed at the top of the screen. Participants are not told according which strategy they are supposed to match the cards, however feedback about whether or not cards were successfully matched is given after each response to facilitate learning. Each participant has 10 trials to learn the correct strategy, and after this the dimension according to which cards have to be matched is changed. Due to this, participants are required to constantly change and adjust to new sorting strategies, and performance can be measured according to several outcomes, e.g. perseverative errors (cognitive flexibility), non-perseverative errors (distractibility), number of trials to complete the first category (conceptual ability), and failure to maintain set (working memory).

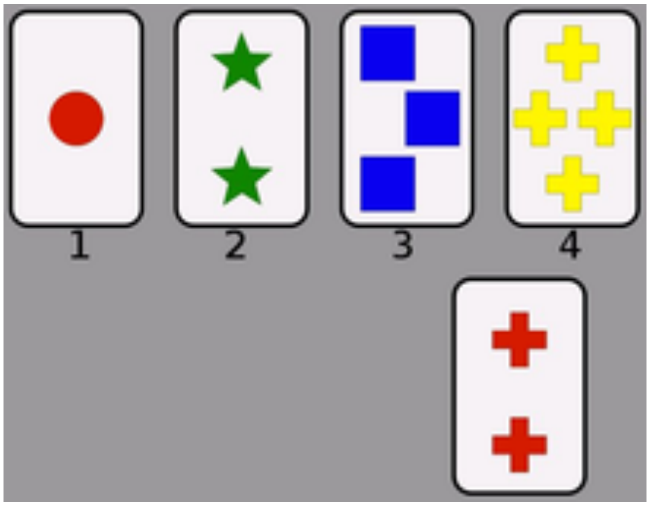

Figure 10. Wisconsin Card Sorting Test sample display

Some investigators, such as Heaton,[19] regard the number of perseverative errors as the most useful measure derived from the WCST. Perseverative errors occur when the subject persists in using a previously successful principle and/or an incorrect matching principle despite feedback indicating that the principle is incorrect. Perseverative errors provide a measure of a subject's sensitivity to contingent feedback.[20] Non-perseverative errors occur when the subject incorrectly sorts the cards without perseverating on the wrong response.[21] The number of non-perseverative errors is typically used as an indicator of distraction.[22,23] The total number of categories achieved reflects overall success, whereas number of trials to complete the first category provides an index of conceptual ability. The failure to maintain set index is a measure of the loss of the correct sorting principle during the course of the testing; this index can be construed as a measure of working memory.[24] The primary outcome of interest we used was the proportion of perseverative errors.

13. Word Fluency

The Controlled Oral Word Association Test (COWAT) was originally developed by Bechtold, Benton et al.,[25] and is a measure of left frontal lobe functioning.[26,27] It tests participants' ability to produce as many words as they can think of that begin with a certain letter (in this study: F, A and S), or belong to a certain category (in this study: animals, fruit/vegetables). The amount of words (no names, people or places; no words with the same root but different ending, e.g. great, greater, greatest) and perservative errors produced within one minute per letter/category gives an indication of left frontal lobe impairments. It has been found to have reasonable test-retest reliability,[28] and updated normative data can be found in Ruff et al. [28] Here only the categories fruit/vegetables and animals are used. The outcome of interest is the average number of correct words produced for both sub-tasks.

14. Digit span task (digit forward task & digit backward task)

The task was taken from Elliott et al.[29] Subjects were asked to listen carefully to a series of random numbers, which were presented at a rate of one per second. In the digit span forwards (DSF), the subject was asked to repeat the numbers in a forward order, while in the digit span backwards (DSB), the subject was asked to recall the numbers in reverse order. The WAIS-digit span procedure was followed. Each subject was read two lists per digit length, starting with a DST three-number sequence. If the subject failed in the two attempts, the test was interrupted and scored according to the longest series that was achieved. If they managed to repeat at least one of the two lists correctly, two sequences with one additional digit followed. For instance, if a subject had correctly recalled a sequence of five digits in the DSF in either the first or second attempt but then failed in the sequence of six digits, he/ she would get a score of 5 in the DSF. The DSF and DSB were scored separately. A maximum of nine digits were presented in the DSF, and a maximum of eight digits were presented in the DSB. Subjects were not penalized if they corrected themselves, and no time limitations were imposed.

The digits themselves are created randomly, and must adhere to the following rules, outlined in **Box 1** below. The principle outcomes of interest were the number of digits a person could recall.

1) No digit occurs more than twice in a sequence
2) Whenever a digit occurs twice in a sequence, at least one other digit intervenes between the two occurrences,
3) No three digits occur in forward numerical order, such as 6, 7, 8/ 4, 6, 8
4) Sequences having general significance, such as certain dates (e.g., 1, 9, 3, 8) are excluded.

Box 1. ***Digit span rules.***

References

1. Bach M. The Freiburg Visual Acuity test--automatic measurement of visual acuity. *Optometry and vision science : official publication of the American Academy of Optometry.* 1996;73(1):49-53.
2. Schulze-Bonsel K, Feltgen N, Burau H, Hansen L, Bach M. Visual Acuities "Hand Motion" and "Counting Fingers" Can Be Quantified with the Freiburg Visual Acuity Test. *Investigative Ophthalmology & Visual Science.* March 1, 2006 2006;47(3):1236-1240.
3. Wesemann W. Visual acuity measured via the Freiburg Visual Acuity Test (FVT), Bailey Lovie Chart and Landolt Ring Chart. *Klinische Monatsblatter Fur Augenheilkunde.* Sep 2002;219(9):660-667.
4. Treutwein B. Adaptive psychophysical procedures. *Vision Research.* 1995;35(17):2503-2522.
5. Lahav K, Levkovitch-Verbin H, Belkin M, Glovinsky Y, U. P. Reduced mesopic and photopic foveal contrast sensitivity in glaucoma. *Archives of Ophthalmology.* 2011;129(1):16-22.
6. Taylor MM, Creelman CD. PEST - Efficient Estimates on Probability Functions. *Journal of the Acoustical Society of America.* 1967 1967;41(4P1):782-&.
7. Roinishvili M, Chkonia E, Stroux A, Brand A, Herzog MH. Combining vernier acuity and visual backward masking as a sensitive test for visual temporal deficits in aging research. *Vision Research.* 2/23/ 2011;51(4):417-423.
8. Herzog MH, Kopmann S, Brand A. Intact figure-ground segmentation in schizophrenia. *Psychiatry Research.* 2004;129(1):55-63.
9. Herzog MH, Koch C. Seeing properties of an invisible object: Feature inheritance and shine-through. *Proceedings of the National Academy of Sciences.* March 27, 2001 2001;98(7):4271-4275.
10. Roudaia E, Pilz KS, Sekuler AB, Bennett PJ. Spatiotemporal properties of apparent-motion perception in aging. *Journal of Vision.* August 5, 2009 2009;9(8):695.
11. Tibber MS, Guedes A, Shepherd AJ. Orientation Discrimination and Contrast Detection Thresholds in Migraine for Cardinal and Oblique Angles. *Investigative Ophthalmology & Visual Science.* December 1, 2006 2006;47(12):5599-5604.
12. Pilz KS, Bennett PJ, Sekuler AB. Effects of aging on biological motion discrimination. *Vision Research.* 2010;50(2):211-219.
13. Theeuwes J, Kooi FL. Parallel search for a conjunction of contrast polarity and shape. *Vision Research.* 1994;34(22):3013-3016.
14. Simon JR, Small AM. Processing Auditory Information - Interference from an Irrelevant Cue. *Journal of Applied Psychology.* 1969 1969;53(5):433-&.
15. Hommel B. The Simon effect as tool and heuristic. *Acta Psychologica.* 2011;136(2):189-202.
16. Castel AD, Balota DA, Hutchison KA, Logan JM, Yap MJ. Spatial attention and response control in healthy younger and older adults and individuals with Alzheimer's disease: Evidence for disproportionate selection impairments in the simon task. *Neuropsychology.* 2007;21(2):170-182.
17. Hick WE. On the Rate of Gain of Information. *Quarterly Journal of Experimental Psychology.* 1952 1952;4:11-26.
18. Berg EA. A Simple Objective Technique for Measuring Flexibility in Thinking. *The Journal of General Psychology.* 1948/07/01 1948;39(1):15-22.
19. Heaton RK. *A Manual for the Wisconsin Card Sorting Test.* Odessa, FL: Psychological Assessment Resources; 1981.

20. Wagman AMI, Wagman W. On the Wisconsin. In: Walker EF, Dworkin RH, Cornblatt BA, eds. Vol 15. New York: Springer; 1992:162–182.
21. Spreen O, Strauss E. *A compendium of neuropsychological tests: administration, norms and commentary.* New York: Oxford University Press; 1991.
22. Barceló F. Electrophysiological evidence of two different types of error in the Wisconsin Card Sorting Test. *Neuroreport.* 1999;10(6):1299-1303.
23. Rhodes MG. Age-Related Differences in Performance on the Wisconsin Card Sorting Test: A Meta-Analytic Review. *Psychology and Aging.* 2004;19(3):482-494.
24. Gooding DC, Kwapil TR, Tallent KA. Wisconsin Card Sorting Test deficits in schizotypic individuals. *Schizophr. Res.* 1999;40(3):201-209.
25. Bechtoldt HP, Fogel ML, Benton AL. An application of factor-analysis in neuropsychology. *Psychological Record.* 1962;12(2):147-&.
26. Newman LM, Trivedi MA, Bendlin BB, Ries ML, Johnson SC. The Relationship Between Gray Matter Morphometry and Neuropsychological Performance in a Large Sample of Cognitively Healthy Adults. *Brain Imaging Behav.* Jun 2007;1(1-2):3-10.
27. Wood AG, Saling MM, Abbott DF, Jackson GD. A neurocognitive account of frontal lobe involvement in orthographic lexical retrieval: An fMRI study. *Neuroimage.* Jul 2001;14(1):162-169.
28. Ruff RM, Light RH, Parker SB, Levin HS. Benton controlled oral word association test: Reliability and updated norms. *Archives of Clinical Neuropsychology.* 1996;11(4):329-338.
29. Elliott E, Cherry K, Brown J, et al. Working memory in the oldest-old: evidence from output serial position curves. *Mem Cogn.* 2011/11/01 2011;39(8):1423-1434.
